# Interprofessional training in medical education: competency, collaboration, and multi-level analysis across seven governorates, Egypt

**DOI:** 10.1186/s12909-025-07369-3

**Published:** 2025-05-27

**Authors:** Ehab Kamal, Yousra El-Maradny, Lobna A. Elgamal, Mennatallah Ahmed Alnagdy, Marwa Rashad Salem, Rasha Ashmawy

**Affiliations:** 1 Medical Research Division, National Research Center (NRC), Giza, Egypt; 2 Medical Biotechnology Department, Institute of Genetic Engineering and Biotechnology, City of Scientific Research and Technological Applications (SRTA-City), New Borg EL-Arab, Alexandria, 21934 Egypt; 3 Department of Intensive Care (ICU), Gamal Abdel Naser Insurance Hospital, Alexandria, Egypt; 4 Department of Clinical Pharmacy Education Affairs, Directorate of Health Affairs, MoHP, Cairo, Egypt; 5 Department of Public Health and Community Medicine, Cairo University, Cairo, Egypt; 6 Directorate of Health Affairs, Ministry of Health and Population, Alexandria, Egypt; 7 PhD candidate at Medical Research Institute, Alexandria University, Alexandria, Egypt

**Keywords:** Interprofessional education, Collaborative practice, Teaching communication, Self-reflection, Team skills, Safety culture, Collaborative practice

## Abstract

**Background:**

Interprofessional Education (IPE) is essential in improving patient outcomes by promoting teamwork and collaboration among healthcare professionals. This study aimed to assess the impact of IPE on developing clinical competencies and collaborative practices in Egyptian intensive care units (ICUs). The core modules focused on managing antibiotic resistance, venous thromboembolism (VTE), and mechanical ventilation (MV), chosen for their high prevalence in Egyptian ICUs and significant impact on patient outcomes.

**Methods:**

The IPE program was implemented across seven governorates, involving 16 hospitals and multidisciplinary ICU teams. It was conducted in two consecutive four-month waves, each covering the three core modules. Participants included physicians, clinical pharmacists, and ICU nurses. To enhance efficacy, a blended learning approach combined virtual webinars, case-based discussions, and in-person workshops. Data collection included pre- and post-tests, a post-training satisfaction survey, and the Interdisciplinary Education Perception Scale (IEPS) to assess competency improvements.

**Results:**

The IPE program included 157 participants, with 79 in wave 1 and 78 in wave 2. Physicians were mostly male (47.2%) and older (> 40 years), while clinical pharmacists and ICU nurses were younger and predominantly female (89.6% and 75.7%, respectively). For exam performance, clinical pharmacists had the highest excellence rate (22.4%), while ICU nurses had the highest failure rate (40.5%). Post-training, interprofessional competence improved significantly, with physicians showing the greatest competency gains (*p* < 0.05) and clinical pharmacists playing a key role in antibiotic resistance management (*p* = 0.029). Overall satisfaction was high across modules, increasing from 79.8% in Module 1 to 90.5% in Module 3. Higher satisfaction was observed among females (up to 89.5%), participants aged 30–40 (92.6% in Module 2), and those who received sufficient program information (*p* = 0.011), with lecturers median score rated consistently 5.0, while material satisfaction median score varied (4.0–4.8).

**Conclusions:**

The IPE program improved interprofessional collaboration and clinical competency, despite challenges such as participant dropout, scheduling conflicts, and engagement in virtual sessions. By addressing issues like antimicrobial resistance and critical care management, it provides a practical model for improving healthcare outcomes, particularly in resource-limited settings. This program is a preliminary step, with plans to expand to more hospitals in Egypt and conduct further research on its long-term impact on patient outcomes and potential for replication in healthcare systems worldwide.

**Clinical trial number:**

Not applicable.

## Introduction

Quality of care is essential for achieving universal health coverage, as it is defined by effective, safe, and people-centered services that align with evidence-based practices. Moreover, health services must be timely, equitable, integrated, and efficient, addressing needs without delays or disparities [1]. Achieving quality in healthcare means providing services that lead to the best possible outcomes, free from harm, and tailored to a patient’s specific conditions. For example, a quality gap occurs when a patient at risk for a pulmonary embolism doesn’t receive appropriate prophylaxis, resulting in a preventable embolus, or when a patient with sepsis doesn’t receive timely antibiotic treatment, leading to worsened outcomes. Addressing these gaps is crucial, as they can cause harm, underscoring the close connection between patient safety and high-quality care [2]. Additionally, critical care relies heavily on effective teamwork, and experts in behavioral sciences emphasize the importance of mastering teamwork skills, especially for leaders, to enhance medical team outcomes during daily rounds and resuscitations [3].

Interprofessional education (IPE) involves students from different healthcare professions learning together to improve collaboration, patient safety, and health outcomes, as defined by the World Health Organization (WHO) [4]. George Thibault of the Josiah Macy Jr. Foundation emphasizes that IPE should complement, not replace, profession-specific education, as each discipline contributes unique expertise to interprofessional collaboration [5]. Several benefits of IPE have been observed, such as increased mutual respect and trust, a deeper understanding of professional roles and responsibilities, enhanced communication, higher job satisfaction, and improvements in patient outcomes, including reduced hospital stays and fewer medical errors [6, 7]. Therefore, IPE is beneficial in ICU settings, where effective teamwork is essential for managing complex, time-sensitive cases, enhances clinical decision-making, patient care, and safety [8].

Although first documented in countries like Canada, the United States, and the United Kingdom, similar initiatives were also emerging independently across the Middle East and North Africa. A 2008 WHO global survey confirmed this trend, with responses from 41 countries demonstrating the widespread adoption of IPE [9]. For example, in the United Kingdom, the introduction of IPE in ICU settings led to a significant reduction in medical errors and improved patient satisfaction [8]. Similarly, successful IPE programs in the United States and Europe have demonstrated positive impacts. The Interprofessional Education Collaborative (IPEC) core competencies framework supports this by emphasizing values and ethics, roles and responsibilities, communication, and teamwork. These successes highlight the potential of IPE to transform healthcare delivery worldwide [10]. Despite these benefits, sustainable implementation of IPE faces challenges such as fragmented curricula across different healthcare professions, a shortage of qualified teaching staff, and limited financial resources. Consequently, most IPE courses remain optional, with only a few being effectively integrated into the curricula of healthcare professionals [11].

In Egypt, IPE has primarily been introduced at the undergraduate level, with universities implementing programs to enhance collaboration among medical, pharmacy, nursing, dentistry, and physical therapy students [12, 13]. These efforts align with the Egyptian Health Council’s 2022 goals for improved teamwork in healthcare. However, little has been done to integrate IPE into hospital settings, particularly in high-pressure environments like ICUs [14]. Barriers such as heavy workloads, resistance to change, and limited resources further challenge its implementation. Addressing these obstacles is crucial to enhance interprofessional collaboration and improving patient care in hospitals [15].

This study aims to bridge this gap by implementing IPE in governmental hospital ICUs and assessing its impact on competency development, collaborative practice, and professional satisfaction. Unlike traditional programs focused on students, this initiative targets practicing healthcare professionals, providing insights into integrating IPE into clinical practice. The findings may inform national strategies for improving patient care in Egypt and serve as a model for other regions facing similar healthcare challenges. This pilot program could be expanded to hospitals nationwide to enhance teamwork and patient outcomes.

## Methodology

### Study design and participants

This longitudinal study evaluated the effectiveness of the IPE program over two waves of participants at governmental hospitals affiliated with the Ministry of Health and Population (MoHP) from July 2022 to October 2023. Wave 1 included hospitals in three northern governorates, while wave 2 covered four southern governorates, aiming to represent Egypt as a first step in the IPE initiative nationwide. These hospitals were selected for their high patient flow, critical care profiles, time-sensitive processes, and complex interprofessional teams. The study involved at least one physician (Internal medicine, critical care, surgery), one clinical pharmacist, and one nurse providing critical care services at these hospitals during the study period, who were willing to participate.

Participant dropouts were closely monitored, with strategies including regular email and phone reminders, flexible scheduling for live sessions, and additional virtual support for those unable to attend in-person. Withdrawn participants’ data was excluded from the final analysis to maintain result integrity.

### Procedure of IPE program development and implementation

The IPE program was meticulously developed and implemented over four months, comprising three core modules focused on improving ICU care in antimicrobial stewardship, venous thromboembolism (VTE) prophylaxis, and mechanical ventilation (MV) management. The blended learning approach was chosen to accommodate the busy schedules of healthcare professionals while providing a mix of theoretical knowledge and practical application. Faculty collaborated with the Egypt Knowledge Bank (EKB) to create a Learning Management System (LMS) (https://mohp.ekb.eg/), where all course materials, including the syllabus, class schedule, patient case information, cultural resources, assignments, and grades, were posted. The program was designed with the integration of various healthcare professionals and supervised by the Egyptian Health Council (https://www.ehc.gov.eg/), which accredited the program. It began with a preparatory phase, during which participants attended an orientation seminar, received access to the LMS, and completed the Interdisciplinary Education Perception Scale (IEPS). This phase also involved the distribution of essential reference materials and initial assessments to establish baseline knowledge.

In Module 1, which spanned two months, participants focused on antimicrobial stewardship. The course content included topics such as antimicrobial prescribing principles, sepsis management, surgical prophylaxis, and combating multidrug-resistant organisms. Participants engaged in nine interactive webinars and completed assignments related to the development of Antimicrobial Stewardship Programs (ASPs). The learning process was supported by various assessment tools, including pre-test and post-test evaluations, multiple-choice questions (MCQs), and team-based projects. Module 2, lasting one month, centered on VTE prophylaxis. Participants were introduced to the fundamentals of blood clot formation, risk factors, prevention strategies, and treatment options, with a particular focus on developing a hospital-specific VTE prophylaxis policy. This module utilized similar instructional and assessment methodologies to Module 1, ensuring continuity in the learning experience.

The final module, also one month long, addressed MV management. Participants explored key aspects of MV, such as indications, modes, patient-ventilator interactions, and respiratory mechanics. The course also covered the prevention of complications, clinical safety, and the role of nursing in MV care. This module culminated in a one-day physical workshop, where participants engaged in case-based discussions, simulation-based learning, and reflective exercises to reinforce their understanding. Throughout the program, a multidisciplinary team of ICU physician consultants, clinical pharmacists, nursing faculty lecturers, and a medical education specialist delivered the content, employing a range of interactive teaching methodologies to ensure a comprehensive and engaging learning experience. The grading system for successful completion of the program is based on several criteria: completing the pre- and post-tests, attending live sessions and recorded webinars, submitting assignments, and finishing the project for each module. Each component is assigned a specific weight in the final grade, which is as follows: Pre- and post-tests (20%), Attendance (Webex Sessions) (25%), Assignments (25%), and Project (30%). The grading scale is as follows: Excellent (≥ 85%), Very Good (≥ 75%), Good (≥ 65%), Fair (≥ 50%), and Fail (< 50%). The exact weight of each component was determined based on its importance in assessing the overall learning outcomes of the program.

### Data collection tools

Data collection included pre- and post-tests, a post-training satisfaction survey, and the Interdisciplinary Education Perception Scale (IEPS). This comprehensive approach provided valuable insights into both the learning outcomes and participant perceptions of the program.

#### Pre- post-test

A self-administered Google Form was used to collect personal data, including gender, specialty, and place of work, along with pre-and post-test questions for the three training modules. The program was delivered through scheduled sessions that combined both theoretical and practical training. Assessment of the training program involved pre- and post-tests, with 150 questions for Module 1, 50 for Module 2, and 57 for Module 3.

#### IEPS

The IEPS pre- and post-questionnaire was administered at the beginning and finish of each IPE wave to evaluate changes in interprofessional attitudes and improvement of IPE competencies. Participants were invited to voluntarily complete an interdisciplinary education perception tool, which is a self-administered questionnaire, previously validated, published in English, and licensed for public use [16]. The tool uses a Likert scale from 1 to 5 (1: strongly disagree, 5: strongly agree). This questionnaire consists of four subscales and 18 items: competence and autonomy are represented in eight items; the perceived need for cooperation is represented in two items; perception for actual cooperation is demonstrated in five items, and lastly understanding others’ value is shown in three items, (supplementary file S1)

#### Satisfaction survey

Additionally, a voluntary, self-administered Google Form was sent to participants after each module to evaluate their satisfaction with the lecturers and training materials. The form also included questions about whether the program required any additional content and what topics they would like future IPE training programs to address.

### Statistical analysis

Descriptive statistics were used to summarize participant demographics and performance metrics. Inferential statistics, including chi-square tests or Fisher’s Exact tests for qualitative data, and the Kruskal Wallis test for quantitative data, were applied to compare satisfaction and performance across different demographic groups and program-related factors. For post hoc analyses, pairwise Z-tests with Bonferroni correction were used for multiple comparisons of proportions, and Dunn’s test with Bonferroni correction was applied for continuous variables. A p-value < 0.05 was considered statistically significant. All analyses were conducted using R version 4.3.3.

## Results

**Fig. 1:**
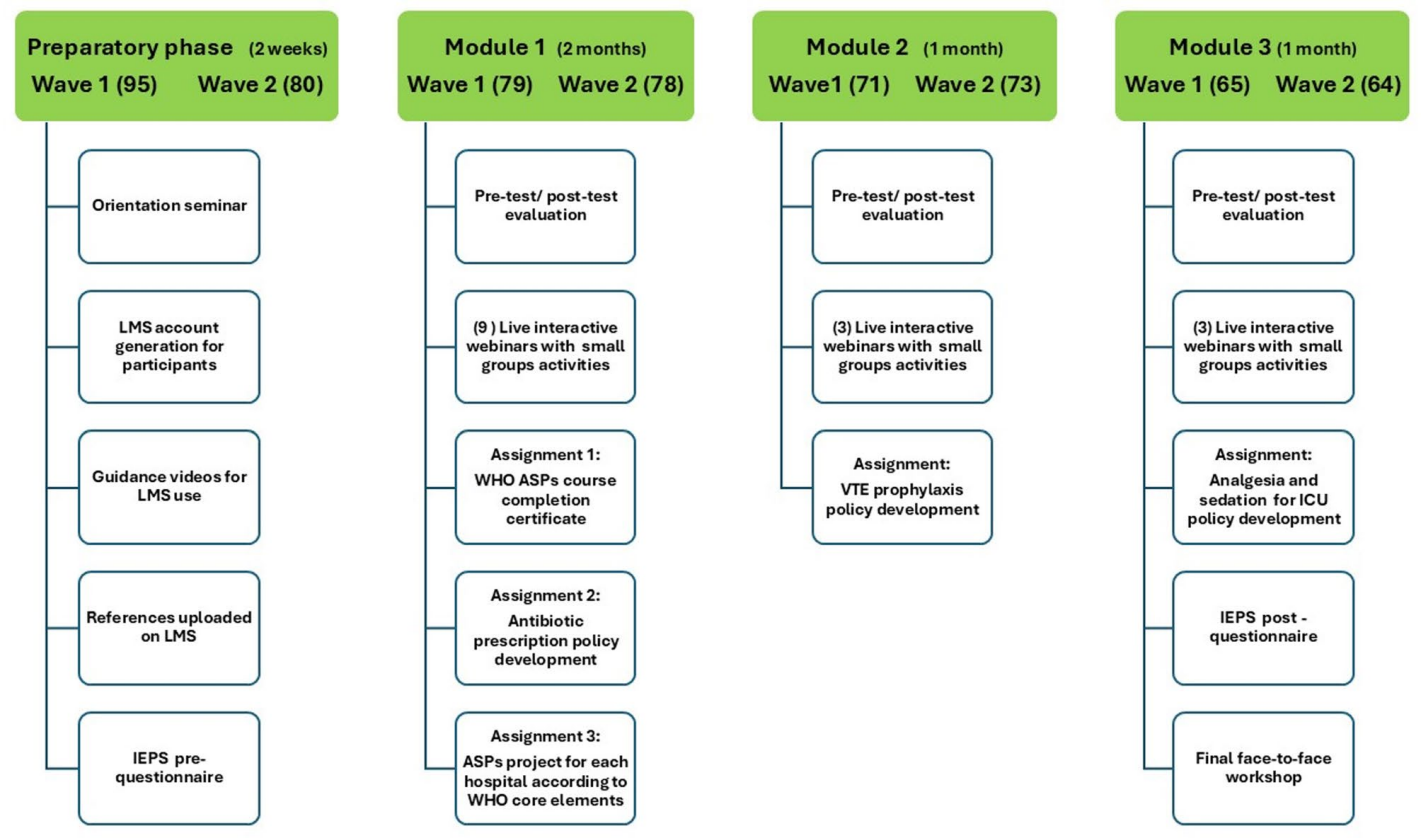
Phased recruitment and educational design of the interprofessional education (IPE) program

Figure 1 illustrates the structured design of the IPE program, detailing the recruitment and educational process across two waves of participants. It begins with a Preparatory Phase (2 weeks), where participants in wave 1 (95) and wave 2 (80) undergo an orientation seminar, receive LMS accounts, and are provided with guidance on using the LMS.

Table 1 provides a comparison of demographic variables among physicians, clinical pharmacists, and ICU nurses in a sample of 157 participants. The majority of participants were aged 30˗40 years, with physicians predominantly older than 40, while clinical pharmacists and ICU nurses were younger. Gender distribution revealed a higher proportion of females, especially among clinical pharmacists (89.6%) and high nurses (75.7%), while nearly half of the physicians were male. Hospital type, governorate, institute affiliation, and wave of the pandemic did not show significant differences across the groups, indicating a relatively consistent distribution across these variables. The participants were almost equally divided between the first (50.3%) and second (49.7%) waves.

**Table 1:** Characteristics of the IPE program participants

Variables	Total (*N* = 157)	Physician (*n* = 53)	Clinical pharmacist (*n* = 67)	ICU nurse (*n* = 37)	*p*-value
Age, years 20–30 >30–40 > 40	43 (27.4%) 81 (51.6%) 33 (21.0%)	5 (9.4%) 29 (54.7%) 19 (35.8%)	24 (35.8%) 34 (50.7%) 9 (13.4%)	14 (37.8%) 18 (48.6%) 5 (13.5%)	0.001*
Gender Female Male	116 (73.9%) 41 (26.1%)	28 (52.8%) 25 (47.2%)	60 (89.6%) 7 (10.4%)	28 (75.7%) 9 (24.3%)	< 0.001*
Hospital General Fever and GIT Oncology Pediatric Pulmonary diseases	114 (72.6%) 13 (8.3%) 6 (3.8%) 9 (5.7%) 15 (9.6%)	38 (71.7%) 4 (7.5%) 1 (1.9%) 4 (7.5%) 6 (11.3%)	50 (74.6%) 5 (7.5%) 2 (3.0%) 4 (6.0%) 6 (9.0%)	26 (70.3%) 4 (10.8%) 3 (8.1%) 1 (2.7%) 3 (8.1%)	0.853
Governorate Alexandria Damietta Giza Assuit Minia Qena Suhag	41 (26.1%) 30 (19.1%) 8 (5.1%) 29 (18.5%) 10 (6.4%) 15 (9.6%) 24 (15.3%)	16 (30.2%) 10 (18.9%) 1 (1.9%) 11 (20.8%) 3 (5.7%) 3 (5.7%) 9 (17.0%)	17 (25.4%) 13 (19.4%) 4 (6.0%) 13 (19.4%) 3 (4.5%) 6 (9.0%) 11 (16.4%)	8 (21.6%) 7 (18.9%) 3 (8.1%) 5 (13.5%) 4 (10.8%) 6 (16.2%) 4 (10.8%)	0.79
Institute Curative sector- MoHP Health Insurance Institute Specialized Medical Centers Institute	126 (80.3%) 11 (7.0%) 20 (12.7%)	44 (83.0%) 4 (7.5%) 5 (9.4%)	53 (79.1%) 5 (7.5%) 9 (13.4%)	29 (78.4%) 2 (5.4%) 6 (16.2%)	0.896
Wave Wave 1 Wave 2	79 (50.3%) 78 (49.7%)	27 (50.9%) 26 (49.1%)	34 (50.7%) 33 (49.3%)	18 (48.6%) 19 (51.4%)	0.973

*Statistically significant with p-value < 0.05

Chi-square or Exact tests were applied for categorical data, represented as a number (%)

Table 2 presents the distribution of exam performance scores across different healthcare professionals, showing significant variations in grade distribution. Although the median scores for Modules 1, 2, and 3 did not differ significantly among the groups (*p* = 0.092, 0.446, and 0.824, respectively), the overall score percentage showed a significant difference (*p* < 0.001). The median score percentage for physicians and clinical pharmacists was 70% (IQR: 60–80%), whereas ICU nurses had a lower median score of 60% (IQR: 30–70%).

**Table 2:** The distribution of exam performance scores across healthcare professionals

Variables	Total (*N* = 157)	Physician (*n* = 53)	Clinical pharmacist (*n* = 67)	ICU nurse (*n* = 37)	*p*-value
Δ Module1, Median (IQR)	61 (8.0, 75.0)	61 (10.0, 75.0)	69 (10.0, 81.0)	53 (5.0, 67.0)	0.092
Δ Module 2, Median (IQR)	30 (12.0, 37.0)	29 (12.0, 35.0)	32 (17.0, 38.0)	27 (9.0, 38.0)	0.446
Δ Module 3, Median (IQR)	24 (1.0, 35.0)	24 (7.0, 36.0)	25 (2.0, 34.5)	20 (0.0, 35.0)	0.824
Score %, Median (IQR)	70% (60%, 80%)	70% (60%, 80%)	70% (60%, 80%)	60% (30%, 70%)	< 0.001*

*Statistically significant with p-value < 0.05, (IQR: Interquartile range)

Kruskal Wallis test was used for numerical data, represented as median (IQR)

**Fig. 2:**
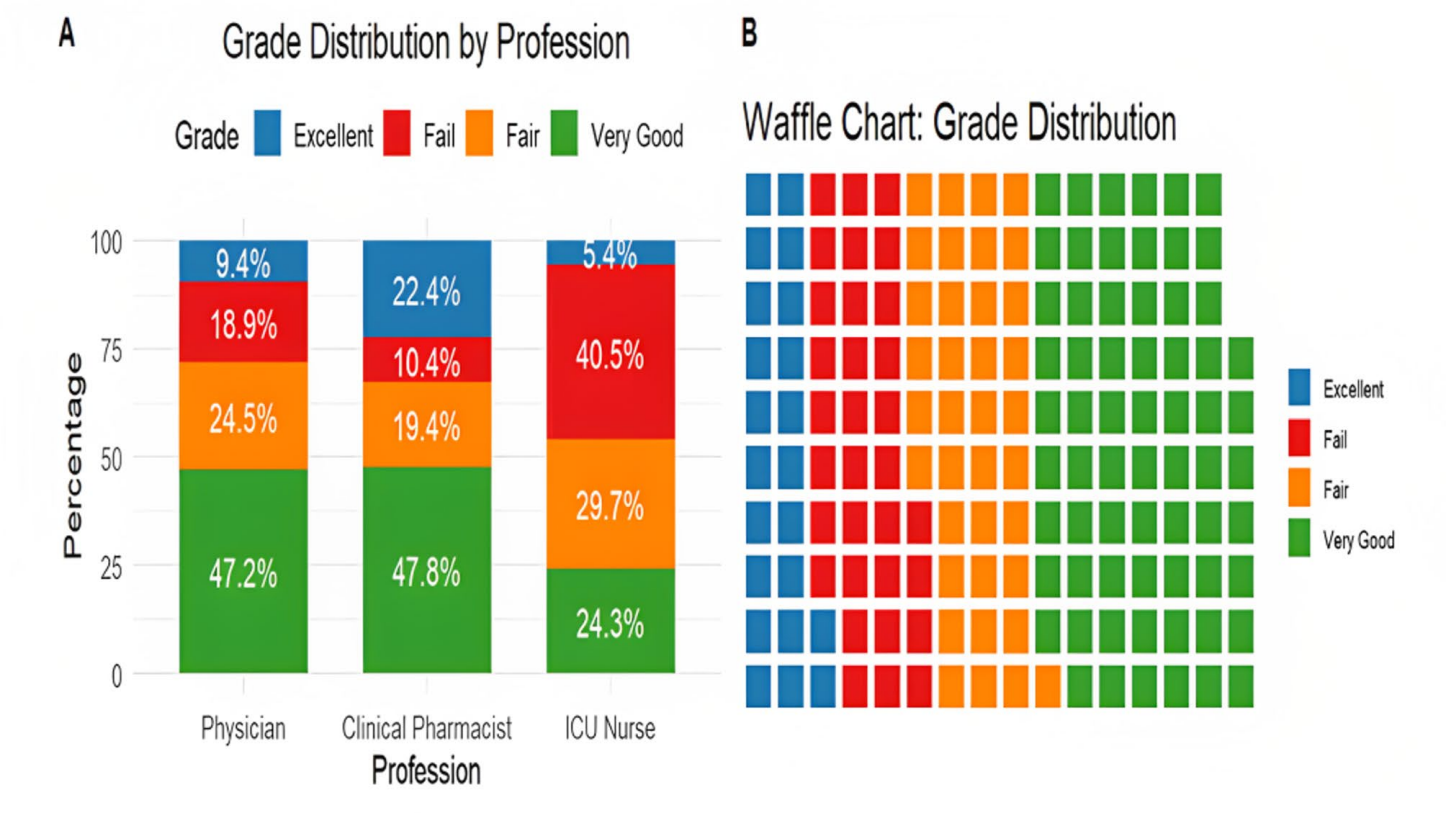
Distribution of categorical grades. **A**) Bar chart comparison by healthcare profession; **B**) Waffle chart of total grade distribution

Figure 2 illustrates the categorical grade distribution among physicians, clinical pharmacists, and ICU nurses using two visualization methods. Panel A presents a bar chart comparing the grade distribution across healthcare professions, showing that “Very Good” was the most common grade among physicians 25 (47.2%) and clinical pharmacists 32 (47.8%), while ICU nurses had a higher proportion of “Fail” grades 15 (40.5%). The “Excellent” grade was most frequent among clinical pharmacists, 15 (22.4%), compared to 5 (9.4%) in physicians and only 2 (5.4%) in ICU nurses. Panel B uses a waffle chart to represent the overall grade distribution, showing that “Very Good” 66 (42.0%) was the most prevalent category, followed by “Fair” 37 (23.6%), “Fail” 32 (20.4%), and “Excellent” 22 (14.0%).

Table 3 demonstrates significant improvements in IEPS survey results across various dimensions of IPE following the training. Pre-training, the competence scores varied significantly among professions, with clinical pharmacists reporting the highest median competence (3.6), while physicians scored the lowest (3.0), nearing statistical significance (*p* = 0.051). Post-training, competence scores increased across all professions, with the greatest improvement seen in physicians (from 3.0 to 4.0), although the differences among the groups were no longer statistically significant (*p* = 0.201). The need for cooperation was recognized by all professions pre-training, with clinical pharmacists indicating the highest median score (4.5), and physicians the lowest (3.0), which was statistically significant (*p* = 0.02). Post-training, the need for cooperation equalized among the groups (*p* = 0.326). Similarly, actual cooperation showed significant improvement, particularly in clinical pharmacists, who reported a substantial increase from 4.2 to 4.5 (*p* = 0.029). Understanding of other professions’ values also improved across the board, though the changes did not reach statistical significance post-training (*p* = 0.781).

**Table 3:** Pre- and post-interprofessional education perception scale (IEPS) survey results across different healthcare specialties

	Item	Total (*N* = 78)	Physician (*n* = 26)	Clinical Pharmacist (*n* = 33)	ICU nurse (*n* = 19)	*p*-value
Pre	Competence and autonomy	3.4 (2.6, 4.1)	3.0 (2.3, 3.5)	3.6 (3.1, 4.3)	3.5 (2.2, 4.2)	0.051
Post		4.0 (3.3, 4.4)	4.0 (3.3, 4.3)	4.1 (3.8, 4.5)	3.7 (2.2, 4.1)	0.201
Pre	Perceived need for cooperation	4.0 (3.0, 4.5)	3.0 (2.6, 4.0)	4.5 (3.5, 4.5)	4.0 (2.2, 4.5)	0.02*
Post		4.0 (3.0, 4.5)	4.5 (3.5, 4.5)	4.0 (3.0, 4.0)	4.0 (2.4, 4.4)	0.326
Pre	Perception of actual cooperation	3.5 (2.8, 4.3)	3.1 (2.7, 3.9)	4.2 (3.2, 4.8)	3.2 (2.1, 4.2)	0.038*
Post		4.0 (3.3, 4.7)	3.9 (3.3, 4.5)	4.5 (4.0, 4.8)	3.5 (2.2, 4.3)	0.029*
Pre	Understanding other values	3.0 (2.5, 4.0)	3.0 (2.1, 3.5)	3.5 (3.0, 4.0)	3.0 (2.5, 4.0)	0.178
Post		3.5 (3.0, 4.0)	3.8 (2.5, 4.5)	4.0 (3.5, 4.0)	3.5 (2.1, 4.4)	0.781

*Statistically significant with p-value < 0.05, (IQR: Interquartile range)

Kruskal Wallis test was used for numerical data, represented as median (IQR)

Overall, satisfaction rates were high across all three modules, with 79.8% of participants satisfied in Module 1, 84.7% in Module 2, and 90.5% in Module 3. Table 4 presents the distribution of satisfaction across three modules, categorized by demographics and program-related factors. Females consistently reported higher satisfaction than males across all modules, accounting for 71.3% of satisfied participants in Module 1, 76.0% in Module 2, and 89.5% in Module 3. The 30–40 age group had the highest satisfaction rates in all modules (84.5%, 92.6%, and 90.9%, respectively), while participants aged 20–30 had lower satisfaction (70.0%, 72.2%, and 66.7%). Regarding professional roles, clinical pharmacists, ICU nurses, and physicians all showed high satisfaction across modules, with no significant differences observed. Participants who received sufficient information before the program reported significantly higher satisfaction, particularly in Module 2 (86.0%, *p* = 0.011), with a significant difference observed between Modules 1 and 2 (*p* = 0.028). In contrast, the mechanism of registration—whether by nomination, announcement, or referral—did not significantly influence satisfaction levels (*p* = 0.191). The majority of participants also felt they were the right fit for the program, with satisfaction rates exceeding 89% across all modules. Satisfaction with lecturers was consistently high, with a median score of 5.0 across all modules, while satisfaction with materials was slightly lower, improving from 4.5 in Module 1 to 4.8 in Module 2 and 4.7 in Module 3, showing statistically significant differences (*p* < 0.001).

**Table 4:** Distribution of satisfaction by demographic characteristics and program-related factors

Variable	Module 1 (*n* = 109)	Module 2 (*n* = 59)	Module 3 (*n* = 21)	*p*-value	Post Hoc
Satisfaction rate	87 (79.8%)	50 (84.7%)	19 (90.5%)	0.432	NA
Gender, female	62/87 (71.3%)	38/50 (76.0%)	17/19 (89.5%)	0.387	NA
Age category 20–30 >30–40 >40	21/30 (70.0%) 49/58 (84.5%) 17/21 (81.0%)	13/18 (72.2%) 25/27 (92.6%) 12/14 (85.7%)	2/3 (66.7%) 10/11 (90.9%) 7/7 (100%)	0.217	NA
Position Clinical Pharmacist ICU Nurse Physician	36/49 (73.5%) 16/20 (80.0%) 35/40 (87.5%)	25/29 (86.2%) 10/12 (83.3%) 15/18 (83.3%)	9/11 (81.8%) 4/4 (100%) 6/6 (100%)	0.856	NA
Sufficient information	61/87 (70.1%)	43/50 (86.0%)	14/19 (73.7%)	0.011*	Module 1 vs. 2: 0.028* Module 1 vs. 3: 0.764 Module 2 vs. 3: 0.284
Mechanism for registration a b c	63/80 (78.8%) 19/22 (86.4%) 5/7 (71.4%)	42/48 (87.5%) 6/7 (85.7%) 2/4 (50.0%)	0/0 19/22 (86.4%) 0/0	0.191	NA
Right person for program	83/87 (95.4%)	48/50 (96.0%)	17/19 (89.5%)	0.598	NA
Satisfaction, lecturers, (Median, IQR)	5.0 (4.7, 5.0)	5.0 (5.0, 5.0)	5.0 (5.0, 5.0)	0.001*	Module 1 vs. 2: 0.001* Module 1 vs. 3: 0.002* Module 2 vs. 3: 0.417
Satisfaction, materials, (Median, IQR)	4.5 (4.0, 5.0)	4.8 (4.3, 5.0)	4.7 (4.2, 5.0)	< 0.001*	Module 1 vs. 2: <0.001* Module 1 vs. 3: 0.001* Module 2 vs. 3: 0.891

*Statistically significant with p-value < 0.05, (IQR: Interquartile range`)

Chi-square or Exact tests were applied for categorical data, representing a number (%), While Kruskal Wallis test was used for numerical data, represented as median (IQR)

Post Hoc: Pairwise Z-tests for proportions, with Bonferroni correction for multiple comparisons for categorical data, Dunn’s Test with Bonferroni correction for numerical data

Mechanisms of registrationa

a. Nomination from the training department in the governorate/authority

b. Register through an announcement on the academy’s pagec

c. Register via Google Form. I received it from a friend

## Discussion

The findings of this study demonstrate the positive impact and effectiveness of the IPE program across healthcare workers in governmental hospitals, particularly among physicians, clinical pharmacists, and ICU nurses. The pre-and post-surveys using the IEPS revealed significant improvements in interprofessional perceptions across all domains, including team-based competencies, autonomy, the perceived need for cooperation, collaboration, and communication. However, the degree of improvement varied across different specialties, highlighting the unique dynamics within each professional group.

The program’s design, consisting of four phases, including a preparatory phase and three core modules, was instrumental in promoting collaborative learning. Starting with 175 participants, the number slightly decreased to 157 after the preparatory phase, possibly due to the participant’s understanding of the program’s curriculum, the commitment required, and the mandatory initial survey. Despite some dropouts, participant attrition between modules was minimal, indicating overall strong engagement throughout the program. Implementing IPE in Egypt comes with several challenges. Limited resources, high patient loads, and a lack of time are some of the key obstacles. Additionally, strong professional identities across disciplines and a sense of loyalty to specific groups further complicate collaboration. The shortage of well-trained and motivated staff adds to these difficulties. Finally, resistance to change, often driven by outdated attitudes, makes the process even more challenging [17, 18].

The representation of pharmacists was notably higher than other specialties, which could be attributed to the larger number of pharmacists in Egypt’s public healthcare sector. According to 2020 statistics [19], pharmacists make up a significant portion of the healthcare workforce in Egypt. Additionally, the significant participation of female healthcare workers, particularly among pharmacists and nurses, aligns with reports from the WHO, which indicate that females constitute approximately 80% of pharmacists and 90% of nurses in Egypt [20]. The majority of participants were from general hospitals, with Alexandria having the highest representation among the seven included governorates: Alexandria, Damietta, Giza, Assuit, Minia, Qena, and Suhag. Alexandria and Giza were urban governorates while a high percentage of the rural population was present in the other governorates. Delivering collaborative healthcare services in rural and high-needs areas presents well-documented challenges [21]. These regions often face additional barriers, such as increased workloads and a shortage of physicians and nurses [22]. In urban areas, the MoHP targets coverage of about 50% of the population, while in rural areas, the target is 100% [23]. These demographic and healthcare infrastructure differences may impact healthcare professionals’ capacity to fully engage with and commit to the program, potentially affecting overall participation and engagement [24].

Exam performance data revealed that pharmacists performed the best across the three modules, followed by physicians and then nurses. Interestingly, Module 1 (focused on antimicrobial stewardship) saw the highest exam performance, possibly reflecting participants’ initial enthusiasm and commitment to the program, which slightly diminished in later modules. The majority of participants achieved grades in the “very good” category, with clinical pharmacists displaying the highest proportion of excellent grades. However, a significant portion of nurses 29.7% received fair grades, and 40.5% failed to complete the program. This performance disparity may be linked to differences in educational backgrounds, prior exposure to similar training, and the relevance of the content to their respective professional roles. Nurses, in particular, may not be as continuously engaged in academic study as other healthcare professionals, potentially contributing to their lower performance. Additionally, challenges in collaboration may have arisen due to differences in attitudes and prior experiences [25]. To support underperforming groups like nurses, it is crucial to provide tailored interventions. For instance, creating flexible training schedules or offering various learning modules could accommodate their demanding workloads. Furthermore, a recent meta synthesis highlights five key themes on continuing professional development (CPD), emphasizing its role in enhancing professionalism, patient care, and competency, while stressing the need for accessible, relevant, and adequately funded CPD [26].

The IEPS survey results further emphasized the program’s effectiveness in improving interprofessional collaboration. Competence scores, in particular, showed a marked increase post-training, with physicians exhibiting the most substantial improvement. This is a promising outcome, as it suggests that the IPE program can help bridge hierarchical gaps that often exist in healthcare settings, enhancing a more collaborative and balanced work environment [27]. Furthermore, the significant increase in the recognition of the need for collaboration and the perception of actual cooperation among all healthcare professionals post-training underscores the program’s success in promoting a culture of teamwork [28, 29]. This outcome aligns with the WHO’s emphasis on interprofessional collaboration as a key strategy for improving patient outcomes and enhancing healthcare quality and safety [30].

The satisfaction survey results indicated a high level of satisfaction across all professional groups and modules, reinforcing the program’s overall success. However, the number of participants completing the satisfaction survey decreased from Module 1 (*n* = 109) to Module 3 (*n* = 21), which suggested a low response rate. This decrease may affect the generalizability of feedback from the later modules and highlights the need to encourage participants to complete the survey, given its importance in informing future program development. Satisfaction with the lecturers and course materials was generally high, with Module 2 and Module 3 participants reporting the highest levels of satisfaction, particularly among those who received clear information and orientation prior to starting the program. The dissatisfaction reported with Module 1 may be due to the content not meeting participants’ expectations, a lack of sufficient depth in the materials, or a disconnect from their professional needs. Clinical pharmacists represented the largest group of respondents, but approximately one-quarter expressed dissatisfaction with Module 1. This could be attributed to pharmacists’ familiarity with ASPs, which forms a core part of their role. As a result, they may have expected more advanced or specialized content than what was provided. Furthermore, a significant portion of participants in Module 1 reported that the curriculum lacked sufficient and comprehensive information compared to the other modules, highlighting a potential gap in meeting the expectations of healthcare professionals with deeper expertise in ASPs and antimicrobial resistance. To improve this, future recommendations incorporate more hands-on examples, interactive activities, and tailored scenarios aligned with participants’ specific roles. Collecting feedback during and after the module would also provide valuable insights to refine the content and ensure it resonates with the audience.

Despite this, the overall satisfaction across modules remained high, particularly among participants who received clear initial information about the program. This emphasizes the importance of clear communication and orientation to ensure that participants understand the program’s structure and objectives, which is crucial for maintaining high engagement and satisfaction levels. Notably, participants who were nominated by their workplaces comprised the majority of those who completed all three modules, and they were the only participants who completed Module 3. This underscores the role that workplace nomination affects commitment and program completion.

In summary, the IPE program proved highly effective in improving interprofessional competencies and increasing collaboration among healthcare professionals in Egyptian governmental hospitals. The improvements observed in performance, attitudes, and satisfaction highlight the potential of IPE to improve teamwork and patient care. However, addressing the performance disparities between professional groups, particularly nurses, and evaluating the long-term impact of the program on clinical practice are important considerations for future research. Expanding such programs across Egypt’s healthcare system could contribute to substantial improvements in healthcare quality and patient safety, aligning with global efforts to promote interprofessional collaboration in healthcare settings. Moreover, sustaining the IPE program in various healthcare settings presents notable challenges. Limited resources—such as funding shortages, high patient-loads, and poor access to online platforms —are major hurdles. Additionally, resistance to change and time constraints among healthcare professionals can hinder further progress.

### Strengths and limitations

This study provides valuable insights, especially with the scarcity of articles addressing the implementation of initiatives aimed at enhancing IPE. Key program elements, such as interactive workshops, team-based assignments, and simulation-based activities, are likely to help participants apply their learning in realistic scenarios. We also consider the limitations of this study, which include the short period of follow-up, the fact that it measured perceptions and attitudes, and the bias resulting from participant dropouts. However, none of these limits the validity of this study. The short-term follow-up captured the immediate and short-term program effects, important features in understanding initial engagement and effectiveness. Perceptions and attitudes have been accepted as key precursors for measures in IPE research. Additionally, comparison of dropouts versus those who remained in the study revealed no significant differences, thereby minimizing selection bias. Whereas future studies are needed to examine longer-term outcomes and direct measures of behavior, the results of this study provide important contributions to the evidence on the effectiveness of the IPE program.

## Conclusion and future directions

In conclusion, this study highlights the effectiveness of a structured IPE program in enhancing interprofessional competencies, attitudes, and collaboration among healthcare professionals in Egyptian governmental hospitals. While various healthcare professionals in a primary healthcare setting are important, it does not inherently ensure effective IPE. It is, therefore, essential to create environments that actively encourage IPE and promote collaborative behaviors across diverse healthcare settings. Additionally, longitudinal studies are needed to evaluate the sustainability of the improvements noted in this study and their long-term effects on clinical practice. Addressing the performance disparities identified between different professional groups, particularly in terms of education and engagement, will be critical in refining future IPE initiatives. Expanding such initiatives could improve healthcare quality and patient safety, particularly in regions where collaboration is vital to overcoming systemic challenges.

## Data Availability

Data will be available upon request from the corresponding author.

## Abbreviations

Δ: Delta
ASPs: Antimicrobial stewardship
GIT: Gastrointestinal Tract
HCPs: Health Care Professionals
ICU: Intensive Care unit
IEPS: The Interdisciplinary Education Perception Scale
IPE: Interprofessional Education
IPEC: The Interprofessional Education Collaborative
IQR: Interquartile range
MoHP: Ministry of Health and Population
MV: Mechanical Ventilation
WHO: World Health Organization
VTE: Venous Thromboembolism
